# VEGF-C Is a Thyroid Marker of Malignancy Superior to VEGF-A in the Differential Diagnostics of Thyroid Lesions

**DOI:** 10.1371/journal.pone.0150124

**Published:** 2016-02-22

**Authors:** Kosma Woliński, Adam Stangierski, Ewelina Szczepanek-Parulska, Edyta Gurgul, Bartłomiej Budny, Elzbieta Wrotkowska, Maciej Biczysko, Marek Ruchala

**Affiliations:** 1 Department of Endocrinology, Metabolism and Internal Medicine, Poznan University of Medical Sciences, Poznan, Poland; 2 Department of General, Gastroenterological and Endocrine Surgery, Poznan University of Medical Sciences, Poznan, Poland; University of Naples Federico II, Naples, Italy, ITALY

## Abstract

**Introduction:**

Thyroid nodular goiter is one of the most common medical conditions affecting even over a half of adult population. The risk of malignancy is rather small but noticeable–estimated by numerous studies to be about 3–10%. The definite differentiation between benign and malignant ones is a vital issue in endocrine practice. The aim of the current study was to assess the expression of vascular endothelial growth factor A (VEGF-A) and VEGF-C on the mRNA level in FNAB washouts in case of benign and malignant thyroid nodules and to evaluate the diagnostic value of these markers of malignancy.

**Materials and Methods:**

Patients undergoing fine-needle aspiration biopsy (FNAB) in our department between January 2013 and May 2014 were included. In case of all patients who gave the written consent, after ultrasonography (US) and fine-needle aspiration biopsy (FNAB) performed as routine medical procedure the needle was flushed with RNA Later solution, the washouts were frozen in -80 Celsius degrees. Expression of VEGF-A and VEGF-C and GADPH (reference gene) was assessed in washouts on the mRNA level using the real-time PCR technique. Probes of patients who underwent subsequent thyroidectomy and were diagnosed with differentiated thyroid cancer (DTC; proved by post-surgical histopathology) were analyzed. Similar number of patients with benign cytology were randomly selected to be a control group.

**Results:**

Thirty one DTCs and 28 benign thyroid lesions were analyzed. Expression of VEGF-A was insignificantly higher in patients with DTCs (p = 0.13). Expression of VEGF-C was significantly higher in patients with DTC. The relative expression of VEGF-C (in comparison with GAPDH) was 0.0049 for DTCs and 0.00070 for benign lesions, medians – 0.0036 and 0.000024 respectively (p<0.0001).

**Conclusions:**

Measurement of expression VEGF-C on the mRNA level in washouts from FNAB is more useful than more commonly investigated VEGF-A. Measurement of VEGF-C in FNAB washouts do not allow for fully reliable differentiation of benign and malignant thyroid nodules and should be interpreted carefully. Further studies on larger groups are indicated. However, measurement of VEGF-C on mRNA level can bring important information without exposing patient for additional risk and invasive procedures.

## Introduction

Thyroid nodular goiter is one of the most common medical conditions affecting–due to numerous studies–even over a half of adult population [1,2]. The risk of malignancy is rather small but noticeable–estimated by numerous studies to be about 3–10% [1,3,4]. The definite differentiation between benign and malignant ones is a vital issue in endocrine practice. The routine evaluation of thyroid lesions encompass ultrasonography (US) and possibly fine-needle aspiration biopsy (FNAB) if the nodule has suspected features–so called sonographic markers of malignancy–in US [5,6,7]. However, as FNAB gives large percentage of inconclusive results (non-diagnostic biopsies, follicular lesions *etc*.), there is a great need for new tools allowing for the reliable pre-surgical diagnosis [8,9]. Numerous techniques, such as elastography, PET/CT or assessment of molecular markers were described in this context [10,11,12,13,14]. The aim of the current study was to assess the expression of vascular endothelial growth factor A (VEGF-A) and VEGF-C on the mRNA level in FNAB washouts in case of benign and malignant thyroid nodules and to evaluate the diagnostic value of these markers of malignancy.

## Materials and Methods

### Patients

The study was approved by The Poznan University of Medical Sciences Ethical Committee. Written informed consent was given by all participants. Patients undergoing fine-needle aspiration biopsy (FNAB) in our department between January 2013 and May 2014 were included.

### Fine-needle aspiration biopsy

In case of all patients who gave the written consent, after US and FNAB performed as routine medical procedure using 25G x 1,5” (0.5 x 40mm) needles. After FNAB the needle was flushed with protective medium—RNA Later solution (Life Technologies). The washouts were immediately frozen in -80 Celsius degrees. Probes of patients who underwent subsequent thyroidectomy and were diagnosed with differentiated thyroid cancer (DTC; proved by post-surgical histopathology) were analyzed. Similar number of patients with benign cytology (patients with colloid nodules were selected) and cancer definitely excluded by at least one-year follow-up or histopathology (in case of patients who underwent thyroid surgery due to other indications than suspicion of malignancy–e.g. big goiter) were randomly selected to be a control group.

### RNA isolation

RNA was isolated with AllPrep Micro Kit (Quiagen) according to the manufacturer’s protocol. The amount of RNA was measured using Qubit 2.0 Fluorometer.

### Reverse transcription

Reverse transcription was performed using RETROscript Reverse Transcription Kit (Life Technologies) according to the manufacturer’s protocol (two step PCR without heat denaturation protocol with the use of random decamers).

### Quantitative PCR

All reactions were performed using StepOne Plus Real-Time PCR System and the primary results were analyzed using StepOne Software (Life Technologies). Expression of VEGF-A, VEGF-C and GAPDH (reference gene) was measured. Experiments were performed using TaqMan Gene Expression Assays (VEGF-A—Hs00900055_m1; VEGF-C—Hs00153458_m1; GAPDH–Hs02758991_g1), TaqMan Gene Expression Master Mix (Life Technologies) and RNase-free water. All experiments were performed in triplicates. Each reaction set involved negative control.

### Statistical analysis

All calculations were performed using Statistica v.10 from Statsoft. The Mann-Whitney U test was used because of the lack of normal distribution of VEGF-A and VEGF-C values in individual groups. The p-value under 0.05 was considered to be statistically significant.

## Results

Thirty one differentiated thyroid cancers (in 31 patients) and 28 benign thyroid lesions (in 25 patients) were analyzed. Among patients with DTCs there were 29 papillary thyroid cancers (PTCs), one follicular thyroid cancer (FTC) and one follicular variant of PTC (fvPTC). According to the TNM classification for thyroid cancer the studied group included 19 patients with pT1, three with pT2, seven with pT3 and two with pT4 stage.

Expression of VEGF-A was insignificantly higher in patients with DTCs. The relative expression of VEGF-A (in comparison with GAPDH) was 0.043 for DTCs and 0.040 for benign lesions, medians – 0.033 and 0.023 respectively (p = 0.13).

**Fig 1:**
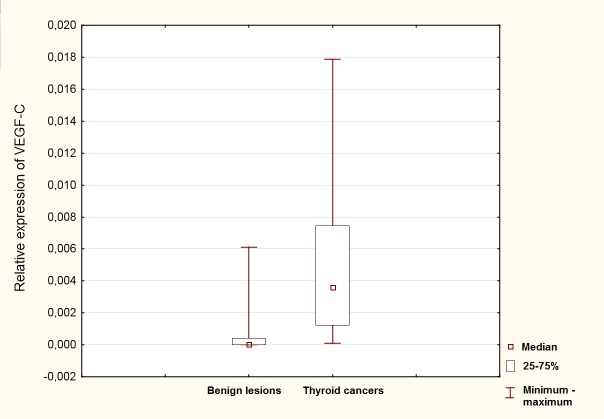
Comparison of relative VEGF-C expression In benign and malignant thyroid nodules.

Expression of VEGF-C was significantly higher in patients with DTC. The relative expression of VEGF-C (in comparison with GAPDH) was 0.0049 for DTCs and 0.00070 for benign lesions, medians – 0.0036 and 0.000024 respectively (p<0.0001; Fig 1). We have also evaluated diagnostic value of particular cut-off points (Table 1).

**Table 1:** Relative expression of VEGF-C in comparison with GAPDH (reference gene)—evaluation of diagnostic value of particular cut-off points.

VEGF-C			
Threshold (relative expression of VEGF-C in comparison to GAPDH)	number of cancers	number of benign lesions	p
7.8*10^−3^ (128 times lower)	7 (22.5%)	0 (0%)	0.01
3.9*10^−3^ (256 times lower)	14 (45.2%)	2 (7.1%)	0.001
9.8*10^−4^ (1024 times lower)	25 (80.6%)	5 (17.9%)	<0.0001
2.4*10^−4^ (4096 times lower)	29 (93.5%)	8 (28.6%)	<0.0001
1.2*10^−4^ (8192 times lower)	30 (96.8%)	9 (32.1%)	<0.0001

## Discussion

Vascular endothelial growth factors constitutes a protein family which was comprehensively studied in context of many neoplasms and other non-neoplastic conditions such as Graves’ disease [15,16]. VEGF-A remains the archetypal and most commonly examined member of the family, which overexpression was described in many types of cancers, especially in the metastatic phase [15,16,17]. Its elevated expression was also described in context of thyroid malignancies both on the RNA and protein level [18,19,20,21,22]. However, according to many of the studies markedly elevated VEGF-A level is typical only for advanced thyroid cancers in the metastatic phase [18,22].

In contrast to VEGF-A being a growth factor for angiogenesis, VEGF-C is mainly involved in development of lymphatic vessels [23]. Overexpression of VEGF-C was also described in context of several neoplasms including thyroid cancer [24,25,26].

According to our results expression of VEGF-A did not differ significantly between DTCs and benign thyroid lesions. Some previous studies revealed overexpression of this factor in case of thyroid malignancies. It is worthy remember that however there were numerous studies about VEGF-A expression in case of thyroid cancers, results varied strongly in the matter of methodology. Giving few examples, in some studies VEGF-A was measured on the protein level [18,19], in other–on mRNA level [27]. Also many different materials were used in previously published studies (e.g. plasma, paraffin-embed thyroid tissues, fresh frozen thyroid tissues, FNAB washouts etc.) [18,19,20,27]. Consequently, results of particular studies can not be directly compared.

Amount of the studies performed with similar methodology–measuring VEGF-A on mRNA level in thyroid tissue—is very limited. According to the study published by Salajegheh et al. in 2011 expression of VEGF-A was higher in PTC in comparison to non-neoplastic thyroid tissues. In patients with classical variant of PTC expression was also higher in those with lymph node metastases [20]. Another study published by the same author in 2013 revealed similar results–overexpression of VEGF-A in PTC and also higher VEGF-A levels in PTCs with metastases [25]. In contrast, Durante et al. did not show significant differences in VEGF-A expression between PTC and normal thyroid tissue [28]. Older study performed by Tanaka et al. using semi-quantitative RT-PCR did not reveal significant differences in expression of VEGF-A between thyroid tissues derived from patients with Graves’ disease and PTC [29].

Some studies has indicated, that overexpression of VEGF-A is rather characteristic for advanced thyroid cancer [18]. The lack of significant difference between DTCs and benign lesions in our study can be partially due to the fact that most of included malignancies were pT1 or pT2 staged.

The amount of studies evaluating diagnostic value of VEGF-C in differential diagnostics of thyroid cancers and benign thyroid lesions is lower than in case of VEGF-A. Available studies also differed in regard to the methodology. Yu et al. [30] described significant elevation of serum VEGF-C on the protein level in patients with PTC; concentrations of the factor were particularly elevated in patients with extrathyroidal invasion or lymph node metastases. According to the retrospective immunohistochemical study performed by Tian et al., VEGF-C expression was present in case of 78.5% of PTCs and 20.0% of adjacent normal thyroid tissues [31]. Another immunohistochemical study performed by Liang et al. revealed higher expression of VEGF-C in case of metastatic cancers in comparison to locally advanced malignancies [32]. Also study performed by Garcia et al. [26] indicated increased lymphatic vessels density and VEGF-C expression at protein level in DTCs. VEGF-C and VEGF-A levels were measured using real-time PCR method by Salajegheh et al. in 2013 [25]. The study revealed overexpression of VEGF-A in over 50% and VEGF-C in 27% of thyroid cancers (mainly PTCs included). The elevation was characteristic mainly for metastatic cancers.

According to our results overexpression of VEGF-C is characteristic for DTCs. Similar results were previously presented by some authors [26,30,31,32]. The current research was performed using washouts from FNAB of thyroid lesions. These fact implicates high clinical importance of the study as it constitutes direct evaluation of diagnostic method, whereas assessment of VEGF-C levels performed on post-surgical thyroid specimens brings results which can be important rather for theoretical contemplations.

In conclusion, measurement of expression VEGF-C on the mRNA level in washouts from FNAB is more useful than more commonly investigated VEGF-A. Measurement of VEGF-C in FNAB washouts do not allow for fully reliable differentiation of benign and malignant thyroid nodules and should be interpreted carefully. Further studies on larger groups are indicated. However, measurement of VEGF-C on mRNA level can bring important information without exposing patient for additional risk and invasive procedures.
